# Lower risk of multiple sclerosis in patients with chronic hepatitis C: a nationwide population-based registry study

**DOI:** 10.1007/s00415-019-09397-8

**Published:** 2019-05-31

**Authors:** Jonas Söderholm, Aylin Yilmaz, Anders Svenningsson, Katharina Büsch, Rune Wejstål, Alma Brolund, Jan Kövamees, Matti Sällberg, Martin Lagging, Magnus Gisslén

**Affiliations:** 1 AbbVie AB, Hemvärnsgatan 9, Solna, Box 1523, 171 29 Stockholm, Sweden; 2 Division of Clinical Microbiology, Department of Laboratory Medicine, Karolinska University Hospital Huddinge, Karolinska Institutet, Stockholm, Sweden; 3 Department of Infectious Diseases, Institute of Biomedicine, Sahlgrenska Academy, University of Gothenburg, Gothenburg, Sweden; 4 Department of Clinical Sciences, Danderyd Hospital, Karolinska Institutet, Stockholm, Sweden

**Keywords:** Hepatitis C, Multiple sclerosis, Registries

## Abstract

**Background:**

Multiple sclerosis (MS) is an immune-mediated neurological disease that causes demyelination. The etiology is unknown, but patients with a previous viral infection, such as Epstein–Barr virus, have been shown to be at a higher risk of developing MS. In contrast, people living with HIV have a lower risk of developing MS. Hepatitis C virus (HCV) mainly infects the liver, but patients with HCV can experience several extrahepatic manifestations and studies have shown an association with several autoimmune conditions such as neuropathy and myelitis. The present study aimed to investigate the risk of MS in patients with chronic HCV infection compared with matched comparators.

**Methods:**

Patients were identified using the nationwide Swedish inpatient (2001–2013) and outpatient care registers (2001–2013) for HCV (B18.2) and MS (G35) according to the International Classification of Diseases-10. Up to five comparators (matched on age/sex/place of residency) were drawn from the general population for each HCV patient. Follow-up started at the first HCV visit from 2001 and the patients’ accrued person-time until death, emigration or 31 December 2013. Risk of MS diagnosis was calculated as standardized incidence ratio (SIR) with 95% confidence intervals (CIs).

**Results:**

HCV patients were at lower risk of MS diagnosis (SIR 0.37; 95% CI 0.26–0.50). The incidence of MS during the study in the HCV cohort was 0.087% compared with 0.27% in the matched comparator cohort.

**Conclusion:**

Surprisingly, these data suggest HCV patients to have a lower risk of MS diagnosis.

**Electronic supplementary material:**

The online version of this article (10.1007/s00415-019-09397-8) contains supplementary material, which is available to authorized users.

## Background

Multiple sclerosis (MS) is an autoimmune disease of the central nervous system, which can lead to both physical and mental symptoms for those affected. The etiology of MS has not been fully elucidated; however, a combination of genetic and environmental factors has been suggested as the underlying cause [1]. Studies have suggested that patients with a previous infection with some viruses, such as Epstein–Barr, measles, rubella, and varicella zoster, are at higher risk of developing MS [2]. In contrast, people living with HIV or are cytomegalovirus (CMV) seropositive have been shown to have a lower risk of developing MS [3–5].

The hepatitis C virus (HCV) is mainly hepatotropic, but has also been reported to infect other cells such as B-cells and monocytes [6, 7]. The HCV entry receptor CD81 is not uniquely expressed on hepatocytes. It is also expressed on B cells as part of a costimulatory complex, and patients with HCV-related lymphoproliferative disorders have elevated serum concentrations of B-cell-activating factor (BAFF) [8]. The HCV-mediated B-cell proliferation leads to the production of monoclonal and polyclonal autoantibodies that has been implicated as the underlying cause of several extrahepatic manifestations [9]. For example, the HCV-induced activation of B-cells generates autoantibodies that can cause mixed cryoglobulinemia, which has been suggested to increase the risk for peripheral neuropathy [10]. Similarly, B-cell proliferation has been proposed as an underlying cause of MS [11], which is why B-cell depletion by anti-CD20 antibody treatment is used as MS therapy [12].

Furthermore, patients with HCV and mixed cryoglobulinemia have increased serum concentrations of the antiganglioside antibody anti-GM1 [10], which has been implicated in Guillain–Barré syndrome as well as in chronic inflammatory demyelinating polyneuropathy [13]. A similar elevation of serum anti-GM1 antibodies has also been observed in patients with MS [14]. Moreover, it has been suggested that HCV induces autoreactive T-cells that are involved in neurological extrahepatic manifestations [9]. In addition, it has been shown that certain single-nucleotide polymorphisms (SNPs) in the Stat1 and in the interferon-α receptor (IFNAR-1) genes are more common both in patients with HCV and in patients with MS compared with controls [15]. These commonalties between patients with HCV and patients with MS, as well as one of the major risk factors for developing MS is smoking [16] and smoking being more common in patients with HCV [17], could suggest patients with HCV to be more prone to develop MS compared with HCV-negative individuals. Nevertheless, the risk for patients with chronic HCV of being diagnosed with MS has not been investigated. The aim of this registry study was to investigate the risk of MS in patients with chronic HCV infection compared with comparators from the general population.

## Methods

### Setting

In Sweden, universal access to healthcare is provided to the population through a tax-funded system. Patients with HCV infection are typically cared for by specialists in infectious diseases or gastroenterology in hospital-based outpatient clinics or inpatient facilities. They are not managed by general practitioners in primary care settings [18]. Likewise, patients with MS are almost exclusively cared for by specialists in neurology. The prevalence of chronic HCV infection in Sweden is estimated to be approximately 0.4% [18] and the prevalence of MS is estimated to be 0.2% [19].

### Data sources and study populations

The Swedish National Patient Register (NPR) is kept by the Swedish National Board of Health and Welfare [20] and contains all inpatient (1987–2013) and non-primary outpatient care (2001–2013) visits, but not primary care visits. The NPR was used to identify patients with chronic HCV infection using the International Classification of Diseases, 10th revision (ICD-10; 2001–2013) code B18.2. Chronic HCV is defined as two positive HCV RNA serum samples at least 6 months apart. Patients with MS were identified as ≥ 1 listing of G35 (ICD-10), with additional requirements for MS diagnosis added to the sensitivity analysis (described below). Information on age, sex, and place of residence, as well as dates of birth and emigration status, was retrieved from the Register of the Total Population held by Statistics Sweden (up to 31 December 2013), which covers the entire Swedish population [21]. Information regarding death was retrieved from the Cause of Death Registry [20]. The highest attained education was retrieved from the Longitudinal Integration Database for Health Insurance and Labour Market Studies (LISA) registry [21]. The Swedish personal identity number (i.e., social security number) was used to link individuals between registers before anonymization. Up to five general population comparators were matched by age, sex, and county of residence to each patient with HCV at the time of HCV diagnosis/identification (the index date) by Statistics Sweden. The study was approved by the Regional Ethics Committee, Karolinska Institutet, Stockholm, Sweden.

### Observation time

The NPR began to include non-primary outpatient care data from 1 January 2001; thus, this date was used as the starting point in the present study. The observation time for the HCV cohort began at the time of the first physician visit, with an accompanying HCV ICD-10 code from 2001 through 2013. These index dates were also used for each comparator. The observation time ended at the time of death, emigration, or 31 December 2013, whichever came first (including time after first MS visit).

### Assessments

The risk for MS diagnosis was expressed using standardized incidence ratios (SIRs), with 95% confidence intervals (CIs), where the number of observed events during the observation time was divided by the number of expected events during the observation time in the HCV cohort based on the events per person-years in the comparator cohort.

### Sensitivity analyses

To reduce the risk of misdiagnosis or entry errors into the registry, the risk for MS diagnosis was also assessed by requiring ≥ 2 listings of MS in the Swedish NPR or by ≥ 2 listings of MS over more than 1 year. In addition, a visit to a neurologist was required for a patient to be considered to have MS (categorized as a visit to a department of Neurology in the NPR). Last, it was required for MS to have been the main diagnosis during the visit to a physician.

### Differential MS diagnosis

Acute disseminated encephalomyelitis (G04.0), neuromyelitis optica spectrum disorders (G36.0), demyelinating disease of the central nervous system CNS (G37.0), acute transverse myelitis not otherwise specified (G37.3), and optic neuritis (H46) are differential diagnoses to MS and the risk for these diagnoses in patients with HCV was also evaluated.

### Statistical methods

Data handling was performed using SAS version 9.4 (SAS Institute Inc., Cary, NC, USA); data analyses were performed using SPSS version 24 (IBM Corp, Armonk, NY, USA). The risk for MS diagnosis was analyzed using SIRs and was considered significant if the 95% CI did not include 1.

## Results

### Lower prevalence and risk of MS diagnosis for patients with HCV infection

**Fig. 1:**
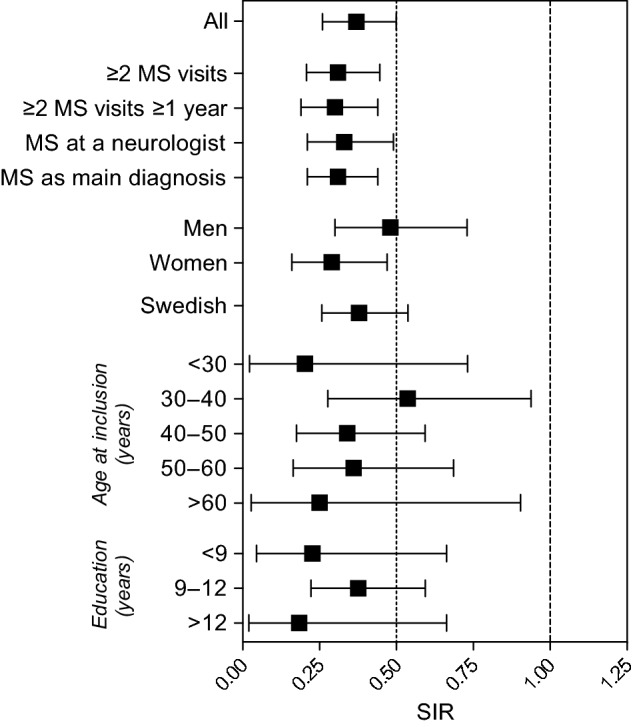
Standardized incidence ratios (95% CI) for being diagnosed with MS in patients with HCV, for all patients, as well as in different subgroups. *HCV* hepatitis C virus, *MS* multiple sclerosis, *SIR* standardized incidence ratio

The total number of patients with an MS diagnosis during the observation time in the HCV patient cohort was 37 of 42,522 (0.087%). The total number of patients with an MS diagnosis during the observation time in the matched comparator cohort was 544 of 202,694 (0.27%). The comparator cohort was followed for 1,504,765 person-years, resulting in 36.2 MS diagnoses per 100,000 person-years. The HCV cohort was followed for 280,123 person-years, and based on the incidence in the comparator cohort, the expected number of patients with HCV infection with an MS diagnosis would be 101 patients from 2001 through 2013. This suggests that patients with HCV were at a lower risk of being diagnosed with MS (SIR 0.37; 95% CI 0.26–0.50; Fig. 1). A retrospective post hoc analysis with a 0.05 two-sided significance level showed a > 99.9% power to detect the difference in incidence between the cohorts.

### Demographics of MS patients

The incidence and prevalence of MS vary in different parts of the world, with Sweden having both a higher incidence and prevalence compared with most other countries [19, 22]. In the present study, around 80% of patients in both the HCV and the comparator cohorts were born in Sweden. The proportion of individuals with a lower level of education was higher in the HCV cohort (Table 1). The mean age at the time of first MS visit during the observation time was 46 years in the HCV cohort and 49 years in the comparator cohort. The mean age at HCV diagnosis was 44 years. The proportion of men was 66.0% in the HCV cohort and 65.5% in the comparator cohort (the ratio of patients with HCV infection to comparators was 4.73 for men and 4.84 for women). The proportion of men with MS was 56.8% in the HCV cohort and 44.1% in the comparator cohort. Among men with HCV infection, 0.075% (21 of 28,072) had an MS diagnosis compared with 0.18% (240 of 132,686) of men in the comparator cohort. For women with HCV infection, 0.11% (16 of 14,450) had an MS diagnosis compared with 0.43% (304 of 70,008) in the comparator cohort. Five percent (*n* = 2) of patients with HCV and MS also had an HIV co-infection, which is numerically higher than the 2% of patients with an HIV co-infection (913 of 42,522) in the full HCV cohort (Table 2).

**Table 1:** Characteristics of the HCV and comparator cohorts

	HCV cohort (*n* = 42,522)	Comparator cohort (*n* = 202,694)
Sex (male)	28,072 (66.0%)	132,686 (65.5%)
Dead	7899 (18.6%)	7516 (3.7%)
Person-years	280,123	1,504,765
Mean observation time (years [95% CI])	6.59 (6.55–6.63)	7.42 (7.41–7.44)
Patients with MS visits during the observation time	37 (0.087%)	544 (0.27%)
MS per 100,000 person-year	13.2	36.2
Expected MS cases (2001–2013)	101	N/A
MS SIR (95% CI)	0.37 (0.26–0.50)	N/A
Land of origin *n* (%)		
Sweden	33,970 (79.9%)	162,387 (80.1%)
Northern Europe	2282 (5.4%)	7878 (3.9%)
Asia	2195 (5.2%)	13,250 (6.5%)
Eastern Europe	1963 (4.6%)	9599 (4.7%)
Africa	1000 (2.4%)	3271 (1.6%)
Western Europe	368 (0.9%)	2154 (1.1%)
Latin America	320 (0.8%)	2222 (1.1%)
South Europe	260 (0.6%)	1098 (0.5%)
North America	140 (0.3%)	713 (0.4%)
Oceania	23 (0.1%)	108 (0.1%)
Information missing	1 (< 0.1%)	14 (< 0.1%)
Highest attained education,^a^*n* (%)		
< 9 years	12,821 (37%)	33,552 (18%)
9–12 years	17,799 (52%)	103,056 (54%)
> 12 years	3611 (11%)	53,202 (28%)

*HCV* hepatitis C virus, *MS* multiple sclerosis, *N/A* not applicable, *SIR* standardized incidence ratio

^a^Highest education available for 34,231 patients HCV infection and 189,810 comparators

**Table 2:** Demographic of patients with MS

	HCV cohort (*n* = 37)	Comparator cohort (*n* = 544)
Dead	7 (19%)	39 (7%)
Emigrated	0	8 (1%)
Sex (male)	21 (57%)	240 (44%)
First MS visit during study (age)^a^	46.3	48.5
HCV diagnosis (age)	43.7	N/A
HIV^+^	2 (5%)	0 (–)
Land of origin, *n* (%)		
Sweden	31 (84%)	454 (84%)
Northern Europe	3 (8%)	25 (5%)
Western Europe	1 (3%)	5 (< 1%)
Eastern Europe	1 (3%)	20 (4%)
Southern Europe	1 (3%)	4 (< 1%)
Asia	0 (–)	26 (5%)
Latin America	0 (–)	4 (< 1%)
Africa	0 (–)	3 (< 1%)
North America	0 (–)	3 (< 1%)

*HCV* hepatitis C virus, *MS* multiple sclerosis, *N/A* = not applicable

^a^The first visit during the study with an MS diagnosis. This may not be the initial MS diagnosis

### Subgroup analysis

The lower risk for patients with HCV infection to have a physician visit with an MS diagnosis remained significant for both men (SIR 0.48; 95% CI 0.30–0.73) and women (SIR 0.29; 95% CI 0.16–0.47), as well as for age at inclusion (< 30 years SIR 0.29; 95% CI 0.16–0.47: 30–40 years SIR 0.54; 95% CI 0.28–0.94: 40–50 years SIR 0.34; 95% CI 0.18–0.59: 50–60 years SIR 0.36; 95% CI 0.16–0.68: > 60 years SIR 0.38; 95% CI 0.25–0.54) The lower risk was also seen in patients with HCV infection who were of Swedish origin (SIR, 0.38; 95% CI, 0.26–0.54) and regardless of highest attained education (< 9 years SIR, 0.23; 95% CI 0.05–0.66: 9–12 years SIR, 0.38; 95% CI 0.22–0.59: > 12 years SIR 0.18; 95% CI 0.02–0.66; Fig. 1).

### Sensitivity analyses

To test the robustness of the SIR analysis, four different requirements were introduced. The risk for MS remained lower after (1) introducing a requirement for ≥ 2 physician visits with an accompanying MS diagnosis (SIR 0.31; 95% CI 0.21–0.45), (2) introducing a requirement for ≥ 2 physician visits over ≥ 1 year with an accompanying MS diagnosis (SIR 0.30; 95% CI 0.19–0.44), (3) introducing a requirement for an MS visit to a neurologist (SIR 0.33; 95% CI 0.21–0.49), or (4) introducing the requirement for a physician visit with MS as the primary diagnosis (SIR 0.31; 95% CI 0.21–0.44; Fig. 1).

### Differential MS diagnoses

Multiple sclerosis is a complex disease and MS-like symptoms can present as other non-MS diseases. Several differential MS diagnoses were, therefore, evaluated to investigate the risk for patients with HCV for these diagnoses. The incidences of these diagnoses were rare in both cohorts with similar risk between the groups (Supplemental Table 1).

## Discussion

Surprisingly this registry study showed that individuals with HCV infection had a 63% lower risk of MS diagnosis compared with a matched comparator cohort. The lower risk for patients with HCV infection to be diagnosed with MS remained after introducing several criteria that improved the stringency of the MS diagnosis by strongly reducing the risk of registration errors. This lower risk of MS diagnosis would make having chronic HCV infection among the highest reported protective factors compared with other previously described factors, including non-smoking, gene markers, HIV infection, vitamin D, or no previous exposure to other viruses such as Epstein–Barr [3, 23, 24]. While MS is more common in women, HCV infection is more common in men. Thus, the MS prevalence in the present study is not representative of the general population. Nevertheless, the lower risk for an MS diagnosis was significant in both men and women.

One possible explanation for the lower risk of MS diagnosis in patients with HCV infection could be that patients with MS are less likely to use illicit injectable drugs, which would reduce the likelihood of HCV exposure. However, a previous study suggested that MS patients are at a higher risk of using illicit drugs, possibly due to depression [25]. Another possibility is that patients with a propensity to develop MS would be more likely to spontaneously resolve an acute HCV infection after initial exposure, and thus, would be less likely to progress to a chronic infection. However, this study was not designed to address these hypotheses.

Smoking has been suggested as an environmental contributor for the development of MS [16]; however, patients with HCV infection are more likely to be smokers compared with HCV-negative controls [17]. As the Swedish registries do not contain any lifestyle factors, the study could not control for smoking. Studies have suggested individuals with higher education to have a lower risk for MS [26, 27], possibly by being a surrogate factor for a healthier lifestyle. The proportion of patients with lower educational level was higher among the patients with HCV, but the lower risk for MS was observed regardless of education. An interesting observation was the possible impact by sex for MS diagnosis between the HCV cohort and the comparator cohort, i.e. there was a smaller difference in frequency of MS diagnosis among men with HCV infection to men in the comparator cohort compared with women for the two cohorts. It was thus a greater reduction of risk for MS diagnosis in women with HCV infection than in men with HCV infection. This could imply that the possible protective factor from HCV for MS development is more prominent in women. In relation to HCV, women are more likely to spontaneously clear the acute HCV infection and to have a slower disease progression [28].

Historically, patients with MS have been treated using interferon. Patients with HCV infection have increased concentrations of systemic type I interferon [29]. In contrast, patients with MS have reduced levels of type I interferon [30]. Thus, one hypothesis for the lower incidence of MS in patients with HCV infection could be the altered immune milieu due to chronic liver infection, in which the elevated systemic type I interferon levels could ameliorate the progression to MS. Interestingly, HIV patients also have chronic immune activation [31], and as previously mentioned, HIV patients are also at a lower risk of developing MS [3, 4].

An obvious confounder to our results that needs to be addressed is possible differences in the genetic susceptibility to HCV and MS. There is a well-known association between MS and the human leukocyte antigen (HLA)-DRB1*15 [23]; however, HLA-DRB1*15 has been reported to be more common in patients developing a persistent HCV infection, as well as in patients with spontaneous clearance [32–35]. Both the studies in European patients showed HLA-DRB1*15 to be associated with viral clearance [33, 35] and could, therefore, in part explain the lower risk for MS in Swedish patients with HCV. In regard to HCV, DRB1*11 has been described as protective for chronicity; however, this allele has also been reported as being significantly underrepresented in patients with MS and, therefore, could not explain our findings [36, 37]. SNPs within or in the vicinity of the interferon lambda 4 (IFNL4; previously referred to as *IL28B* or IFNL3 [38]) gene have been shown to be predictive of patients spontaneously resolving acute HCV infection, as well as patients responding to interferon-α [39, 40]. For MS, the IFNL4 SNPs were not associated with a response to interferon-β [41].

### Strengths and limitations

The main strength of our study is the large number of patients included, with virtually complete national coverage. Registry studies, however, are generally limited by the quality of data entered; however, in this study, both the MS and HCV diagnoses were made by specialists, with HCV by law being a notifiable disease in Sweden [42]. Additionally, the concordance for HCV diagnosis between the NPR and the mandatory communicable disease register has been shown to be very high [43]. With regard to MS, a recent study showed that the Swedish NPR included 99.7% of Swedish patients with MS [22]. The study did not investigate the use of MS or HCV treatment, since information on dispensed drugs was only available from July 2005.

The fact that we have no information regarding the known risk factors for MS (such as smoking, vitamin D levels, and HLA haplotype) for the patients with HCV infection and their comparators imposes a weakness in the interpretation of our data. However, from what is known about these factors in patients with HCV infection, it is unlikely that such factors could explain all of the negative association observed between HCV infection and MS.

In the general population, MS onset normally occurs around 30 years of age, but since the initial age of HCV diagnosis in the present study was 44 years, the cohorts are older than what would have been expected if the study population had instead looked at the initial MS diagnosis. Most patients with MS would, therefore, have been diagnosed with MS before inclusion into the present study. However, most patients with HCV are generally exposed to HCV in their early 20 s [44], whereas initial MS symptoms usually debut at approximately 30 years of age [22, 45]; in other words, the majority of patients would have had chronic HCV well ahead of the common age of MS onset.

In conclusion, this study suggests that MS is less common in patients with chronic HCV infection. Further studies are needed to elucidate the mechanisms and validity behind this novel finding.

## Electronic supplementary material

Below is the link to the electronic supplementary material.

Supplementary file1 (DOCX 14 kb)

## References

[1] Reich DS, Lucchinetti CF, Calabresi PA (2018). Multiple sclerosis. Longo DL, editor. N Engl J Med.

[2] Oskari Virtanen J, Jacobson S (2012). Viruses and multiple sclerosis. CNS Neurol Disord-Drug Targets Former Curr Drug Targets-CNS Neurol Disord.

[3] Gold J, Goldacre R, Maruszak H, Giovannoni G, Yeates D, Goldacre M (2015). HIV and lower risk of multiple sclerosis: beginning to unravel a mystery using a record-linked database study. J Neurol Neurosurg Psychiatry..

[4] Nexø BA, Pedersen L, Sørensen HT, Koch-Henriksen N (2013). Treatment of HIV and Risk of multiple sclerosis. Epidemiology.

[5] Sundqvist E, Bergström T, Daialhosein H (2014). Cytomegalovirus seropositivity is negatively associated with multiple sclerosis. Mult Scler J.

[6] Zignego AL, Macchia D, Monti M (1992). Infection of peripheral mononuclear blood cells by hepatitis C virus. J Hepatol.

[7] Ducoulombier D, Roque-Afonso A-M, Liberto GD (2004). Frequent compartmentalization of hepatitis C virus variants in circulating B cells and monocytes. Hepatology.

[8] Himoto T, Masaki T (2012). Extrahepatic manifestations and autoantibodies in patients with hepatitis C virus infection. Clin Dev Immunol.

[9] Mariotto S, Ferrari S, Monaco S (2014). HCV-related central and peripheral nervous system demyelinating disorders. Inflamm Allergy Drug Targets.

[10] Alpa M, Ferrero B, Cavallo R (2008). Anti-neuronal antibodies in patients with HCV-related mixed cryoglobulinemia. Autoimmun Rev.

[11] Disanto G, Morahan JM, Barnett MH, Giovannoni G, Ramagopalan SV (2012). The evidence for a role of B cells in multiple sclerosis. Neurology.

[12] Torres IM, García-Merino A (2017). Anti-CD20 monoclonal antibodies in multiple sclerosis. Expert Rev Neurother.

[13] Simone IL, Annunziata P, Maimone D, Liguori M, Leante R, Livrea P (1993). Serum and CSF anti-GM1 antibodies in patients with Guillain–Barré syndrome and chronic inflammatory demyelinating polyneuropathy. J Neurol Sci.

[14] Acarin N, Rio J, Fernandez AL (1996). Different antiganglioside antibody pattern between relapsing-remitting and progressive multiple sclerosis. Acta Neurol Scand.

[15] Fortunato G, Calcagno G, Bresciamorra V (2008). Multiple sclerosis and hepatitis C virus infection are associated with single nucleotide polymorphisms in interferon pathway genes. J Interferon Cytokine Res.

[16] Wingerchuk DM (2012). Smoking: effects on multiple sclerosis susceptibility and disease progression. Ther Adv Neurol Disord.

[17] Kim RS, Weinberger AH, Chander G, Sulkowski MS, Norton B, Shuter J (2018). Cigarette smoking in persons living with hepatitis C: the National Health and Nutrition Examination Survey (NHANES), 1999–2014. Am J Med.

[18] Büsch K, Waldenström J, Lagging M (2017). Prevalence and comorbidities of chronic hepatitis C: a nationwide population-based register study in Sweden. Scand J Gastroenterol.

[19] Ahlgren C, Odén A, Lycke J (2011). High nationwide prevalence of multiple sclerosis in Sweden. Mult Scler J..

[20] Swedish National Board of Health and Welfare (Socialstyrelsen) (2016) Information available on the National Patient Registry. http://www.socialstyrelsen.se/SiteCollectionDocuments/information-in-the-national-patient-register.pdf. Accessed Sept 2018

[21] Statistics Sweden (2015) Microdata at Statistic Sweden for research purposes. https://www.scb.se/Grupp/Tjanster/SCBs-data-for-forskning.pdf/. Accessed Sept 2018

[22] Ahlgren C, Odén A, Lycke J (2014). High nationwide incidence of multiple sclerosis in Sweden. PLoS ONE.

[23] Simon KC, van der Mei IAF, Munger KL (2010). Combined effects of smoking, anti-EBNA antibodies, and HLA-DRB1*1501 on multiple sclerosis risk. Neurology.

[24] Salzer J, Hallmans G, Nyström M, Stenlund H, Wadell G, Sundström P (2012). Vitamin D as a protective factor in multiple sclerosis. Neurology.

[25] Bombardier CH, Blake KD, Ehde DM, Gibbons LE, Moore D, Kraft GH (2004). Alcohol and drug abuse among persons with multiple sclerosis. Mult Scler J.

[26] Bjørnevik K, Riise T, Cortese M (2016). Level of education and multiple sclerosis risk after adjustment for known risk factors: the EnvIMS study. Mult Scler Houndmills Basingstoke Engl.

[27] Bjørnevik K, Riise T, Benjaminsen E (2017). Level of education and multiple sclerosis risk over a 50-year period: registry-based sibling study. Mult Scler Houndmills Basingstoke Engl.

[28] Baden R, Rockstroh JK, Buti M (2014). Natural history and management of hepatitis C: does sex play a role?. J Infect Dis.

[29] Mihm S (2015). Activation of type I and type III interferons in chronic hepatitis C. J Innate Immun.

[30] Reder AT, Feng X (2013). Aberrant type I interferon regulation in autoimmunity: opposite directions in MS and SLE, shaped by evolution and body ecology. Front Immunol.

[31] Appay V, Sauce D (2008). Immune activation and inflammation in HIV-1 infection: causes and consequences. J Pathol.

[32] Ksiaa L, Ayed-Jendoubi S, Sfar I (2007). Clearance and persistence of hepatitis C virus in a tunisian population: association with HLA class I and class II. Viral Immunol.

[33] McKiernan SM, Hagan R, Curry M (2004). Distinct MHC class I and II alleles are associated with hepatitis C viral clearance, originating from a single source. Hepatology.

[34] Huang J, Huang K, Xu R (2016). The associations of HLA-A*02:01 and DRB1*11:01 with hepatitis C virus spontaneous clearance are independent of *IL28B* in the Chinese population. Sci Rep.

[35] Lechmann M, Schneider EM, Giers G (1999). Increased frequency of the HLA-DR15 (B1*15011) allele in German patients with self-limited hepatitis C virus infection. Eur J Clin Invest.

[36] Abolfazli R, Samadzadeh S, Sabokbar T (2014). Relationship between HLA-DRB1* 11/15 genotype and susceptibility to multiple sclerosis in Iran. J Neurol Sci.

[37] Cangussu LOF, Teixeira R, Campos EF (2011). HLA class II alleles and chronic hepatitis C virus infection. Scand J Immunol.

[38] Dean L (2012) Sofosbuvir Therapy and IFNL4 Genotype. In: Pratt V, McLeod H, Rubinstein W, Dean L, Kattman B, Malheiro A (eds). Med Genet Summ [Internet]. Bethesda (MD): National Center for Biotechnology Information (US). https://www.ncbi.nlm.nih.gov/books/NBK409960/. Accessed 7 Nov 2018 28520377

[39] Ge D, Fellay J, Thompson AJ (2009). Genetic variation in IL28B predicts hepatitis C treatment-induced viral clearance. Nature.

[40] Thomas DL, Thio CL, Martin MP (2009). Genetic variation in *IL28B* and spontaneous clearance of hepatitis C virus. Nature.

[41] Malhotra S, Morcillo-Suárez C, Brassat D (2011). IL28B polymorphisms are not associated with the response to interferon-beta in multiple sclerosis. J Neuroimmunol.

[42] Duberg A, Janzon R, Bäck E, Ekdahl K, Blaxhult A (2008). The epidemiology of hepatitis C virus infection in Sweden. Euro Surveill Bull Eur Sur Mal Transm Eur Commun Dis Bull.

[43] Hofmann JN, Törner A, Chow W-H, Ye W, Purdue MP, Duberg A-S (2011). Risk of kidney cancer and chronic kidney disease in relation to hepatitis C virus infection: a nationwide register-based cohort study in Sweden. Eur J Cancer Prev.

[44] Kåberg M, Navér G, Hammarberg A, Weiland O (2018). Incidence and spontaneous clearance of hepatitis C virus (HCV) in people who inject drugs at the Stockholm Needle Exchange—importance for HCV elimination. J Viral Hepat.

[45] Svenningsson A, Salzer J, Vågberg M, Sundström P, Svenningsson A (2015). Increasing prevalence of multiple sclerosis in Västerbotten County of Sweden. Acta Neurol Scand.

