# Supplementary material for: Lower risk of multiple sclerosis in patients with chronic hepatitis C: a nationwide population-based registry study
Source: J Neurol. 2019 May 31;266(9):2208–15. doi: 10.1007/s00415-019-09397-8 (PMC6687702; doi:10.1007/s00415-019-09397-8)
Supplement: Supplementary file 1 — Supplementary file1 (DOCX 14 kb) [file 415_2019_9397_MOESM1_ESM.docx]

**Supplemental Table 1 – Differential MS diagnoses**

| Diagnosis | ICD-10 | SIR | Observed | Expected |
| --- | --- | --- | --- | --- |
| Acute disseminated encephalomyelitis | G04.0 | - | 0 | 0 |
| Neuromyelitis optica spectrum disorders | G36.0 | - | 0 | 0.39 |
| Demyelinating disease of the CNS | G37.0 | - | 0 | 0.39 |
| Acute transverse myelitis NOS | G37.3 | 2.05 (95% CI, 0.23-7.41) | 2 | 0.97 |
| Optic neuritis | H46 | 0.55 (95% CI, 0.26-1.01) | 10 | 18.13 |

CNS = Central nervous system; NOS = Not otherwise specified
